# Sensing Stemness

**DOI:** 10.1007/s40778-021-00201-w

**Published:** 2021-10-06

**Authors:** Teresa V. Bowman, Eirini Trompouki

**Affiliations:** 1 Department of Developmental and Molecular Biology, Albert Einstein College of Medicine, Bronx, NY USA; 2 Gottesman Institute for Stem Cell Biology and Regenerative Medicine, Albert Einstein College of Medicine, Bronx, NY USA; 3 Department of Medicine (Oncology), Albert Einstein College of Medicine and Montefiore Medical Center, Bronx, NY USA; 4 Department of Cellular and Molecular Immunology, Max Planck Institute of Immunobiology and Epigenetics, Freiburg, Germany; 5 CIBSS-Centre for Integrative Biological Signaling Studies, University of Freiburg, Freiburg, Germany

**Keywords:** Hematopoietic stem cells, DNA sensors, RNA sensors, R-loops, Transposable elements, Plasticity

## Abstract

***Purpose of Review*:**

Hematopoietic stem cells (HSCs) are formed embryonically during a dynamic developmental process and later reside in adult hematopoietic organs in a quiescent state. In response to their changing environment, HSCs have evolved diverse mechanisms to cope with intrinsic and extrinsic challenges. This review intends to discuss how HSCs and other stem cells co-opted DNA and RNA innate immune pathways to fine-tune developmental processes.

***Recent Findings*:**

Innate immune receptors for nucleic acids like the RIG-I-like family receptors and members of DNA sensing pathways are expressed in HSCs and other stem cells. Even though the “classic” role of these receptors is recognition of foreign DNA or RNA from pathogens, it was recently shown that cellular transposable element (TE) RNA or R-loops activate such receptors, serving as endogenous triggers of inflammatory signaling that can shape HSC formation during development and regeneration.

***Summary*:**

Endogenous TEs and R-loops activate RNA and DNA sensors, which trigger distinct inflammatory signals to fine-tune stem cell decisions. This phenomenon could have broad implications for diverse somatic stem cells, for a variety of diseases and during aging.

## Introduction

Hematopoietic stem cells (HSCs) reside on the top of the hematopoietic hierarchy and can give rise to the majority of differentiated blood cells [1–5]. HSCs arise during development through a highly plastic process involving a dynamic cell fate and morphological transition termed the endothelial-to-hematopoietic transition or EHT [6, 7]. During this process, a specialized fraction of endothelial cells will give rise to hemogenic endothelial cells which later become HSCs. HSCs then proliferate in specified organs such as the fetal liver in mouse and the caudal hematopoietic tissue in zebrafish [8, 9]. During this plastic process, the cells undergo extensive chromatin reorganization [10] and transcriptional reprogramming that are cell-type specific. Global DNA rearrangement is also observed when quiescent adult HSCs enter a transient activated state during times of stress, including during infection, chemotherapy, and after extensive bleeding [4, 11]. Global DNA rearrangement can result in the transcription of transposable elements (TEs) and other repetitive RNAs or the formation of R-loops, comprised of RNA:DNA hybrids and single-stranded (ss) DNA [12–15].

DNA and RNA sensing pathways are abundantly active in immune cells in order to recognize foreign DNA or RNA, but are also used as the first line of defense against pathogens in non-immune effector cells [16, 17]. Nucleic acid sensors include the endosomal Toll-like receptors (TLRs) TLR3, TLR7, TLR8, and TLR9 [18]; the nuclear and cytosolic DNA sensor cyclic GMP–AMP synthase (cGAS) [19, 20]; retinoic acid-inducible gene I (RIG-I)-like receptors (RLRs) [21]; NOD-like receptors (NLRs) [22]; AIM2‐like receptors (ALRs) [23]; and C-type lectins [24]. DNA-sensing pathogen recognition receptors (PRRs) include cGAS, the endosomal TLR9, and the cytosolic ALRs AIM2 and IFI16. RNA-sensing PRRs are endosomal TLR3, TLR7, TLR8, and cytosolic RIG‐I, MDA5, NLRP1 and 3, and NOD2 [17]. Interaction of these receptors with viral RNA or DNA from pathogens transduce innate immune signaling pathways that cause transcriptional activation by nuclear factor kappa B (NF-κB), IFN-response factors (IRF), and other inflammatory transcription factors [16, 17]. Importantly, HSCs and other somatic stem cells express many of these receptors and downstream pathway components resulting in specific expression of interferon-stimulated genes (ISGs) that protects them from viral invasion.

Nucleic acid sensors recognize different types of DNA and RNA species [16, 17]. For example, RIG-I recognizes short 5′ ppp‐double-stranded (ds) RNA while MDA5 prefers long dsRNA. TLR3 recognizes dsRNA, TLR7/8 recognize single-stranded RNA, and TLR9 recognizes unmethylated CpG DNA. Evidence has been accumulating over the last decade demonstrating that endogenous sources of RNAs and DNAs can also stimulate nucleic acid sensors. For example, mislocalization of mitochondrial RNA or DNA in the cytosol stimulates MDA5 and cGAS, respectively, both resulting in induction of a type I IFN response [25, 26]. Mistakes in A-to-I RNA editing by adenosine deaminase acting on RNA 1 (ADAR1) or unedited *Alu* elements are also major sources of non-viral MDA5 stimulation [27–29]. Persisting double-strand breaks, micronuclei formation that occur during mitosis and accumulation of cytosolic DNA are common sources of self-activation of cGAS that lead to non-viral activation of inflammatory signaling [19].

Many pivotal studies have proven the importance of inflammatory signaling on HSCs from birth through old age and under times of stress. Only recently has the initiating, endogenous source of inflammatory signaling in HSCs started to be elucidated. In this review, we will focus mainly on the role that RLR and cGAS nucleic acid sensing pathways play in developmental decisions and stemness. We are focusing particularly on the hematopoietic system but also presenting evidence for a non-pathogenic role of DNA/RNA sensors in other stemness contexts.

### DNA Sensing-cGAS Pathway

**Fig. 1:**
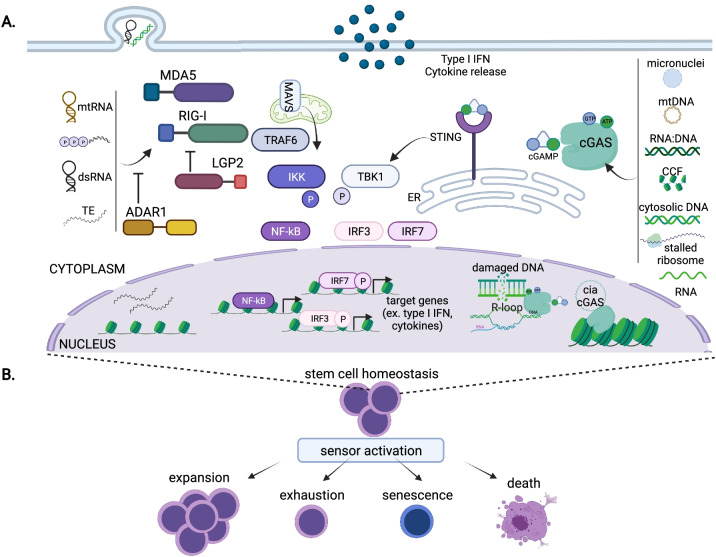
**A** RIG-I-Like receptors (RLRs), such as MDA5 and RIG-I, are activated by numerous cytosolic ligands, such as mitochondrial RNA (mtRNA), 5′-triphosphate RNA, dsRNA, and transposable element transcripts (TEs). cGAS is stimulated in both the cytoplasm and nucleus by several endogenous stimuli, such as micronuclei, mitochondrial DNA (mtDNA), RNA:DNA hybrids, cytosolic chromatin fragments (CCF), naked cytosolic DNA, stalled ribosomes, and most recently RNA. Activation of RLRs promotes oligomerization with the mitochondrial anti-viral signaling protein (MAVS), which triggers activation of kinases like Tank Binding Kinase 1 (TBK1) and IκB kinase ε (IKKε). Stimuli binding to cGAS induces its synthetase activity resulting in production of the second messenger cyclic GMP-AMP (cGAMP). STING activation by cGAMP results in induction of TBK1. Stimulation of either RNA or DNA sensor pathways can ultimately result in the translocation of Interferon Response Factor 3 (IRF3), IRF7, and nuclear factor kappa B (NF-κB) to the nucleus leading to the induction of an IFN response and the secretion of proinflammatory cytokines. Cells possess negative regulators of the sensor pathways including LGP2 and ADAR1 for RLRs and nucleosome tethering and the circular RNA cia-cGAS for nuclear cGAS. **B** Nucleic acid sensor activation alters stem cell homeostasis in cell-type and context-specific manners. Pathway activation can result in diverse cellular outcomes, such as expansion, exhaustion, senescence, or cell death. Created with BioRender.com

cGAS, a recently identified DNA sensor, is thought to be one of the main responders to infectious and damaged self-DNA [19, 20]. cGAS is activated by dsDNA in both the cytoplasm and nucleus in a manner independent of specific DNA sequence [19, 20]. Upon DNA binding, cGAS produces the second messenger 2′,3′-cyclic-GMP-AMP (cGAMP), which in turn binds to and activates the endoplasmic reticulum transmembrane receptor Stimulator of Interferon Genes (STING). Then, STING triggers distinct downstream mechanisms, including activation of Tank Binding Kinase 1 (TBK1); nuclear translocation of the transcription factors IRF3, IRF7, and NF-κB; stimulation of type I IFN gene expression; and production of inflammatory cytokines (Fig. 1) [19, 20].

Although largely explored as a dsDNA sensor, recent studies revealed that cGAS can be activated by a variety of non-DNA stimuli [19]. For example, nuclear cGAS can be relocalized and activated during translational stress due to recognition of unresolved collided ribosomes as a consequence of a disruption of the ribosome-associated protein quality control [30]. Very recently, cGAS-like receptors (cGLRs) were characterized as innate immune receptors in *Drosophila melanogaster* and surprisingly were identified as RNA binders. Crystal structure studies identified differences in the ligand binding surface between cGLR1 and c-GAS that could explain the differences in nucleic acid binding preferences [31, 32]. These new findings highlight that our understanding of all the potential ligands for cGAS as well as what guides the selectivity is likely only the tip of the iceberg.

Depending on the cellular context, these effectors can result in cell proliferation, autophagy, cell senescence, or cell death [33–39]. Activation of cGAS during mitotic arrest results in accumulating, low-level IRF3 phosphorylation without inflammatory gene upregulation, but rather suppression of BCL-XL and subsequent apoptosis [39]. Additionally, STING activation can trigger autophagy via a non-canonical pathway resulting in STING translocation to the ER-to-Golgi interface and ultimately LC3 lipidation [34]. Lysosome-associated, activated STING can also trigger cell death by promoting membrane permeabilization [35]. Furthermore, exposure of mice to the STING agonist carboxymethyl-9-acridanone (CMA) results in robust pathway activation in CD4 + T cells, which promotes expression of a distinct gene set and ultimately results in apoptosis [40]. In contrast, cGAS-STING signaling in tumor-infiltrating CD8 + T cells enhances the formation of stem-like memory T cells [41]. Signal strength and cell type differences are suggested to impart these distinct cellular outcomes [40]. There is still much to learn about the repertoire and regulation of cGAS-STING signaling and downstream outcomes.

### cGAS-ing Stemness

Although mostly studied in immune effector cells, cGAS-STING signaling components are broadly expressed, comprising an ancient frontline defense of innate immunity. Several recent findings directly demonstrate that the cGAS-STING pathway is critical for developmental and adult hematopoietic stem and progenitor cell (HSPC) homeostasis even though this field is still in its infancy.

Many years ago, it was demonstrated that polymicrobial intraperitoneal infection in mice promotes HSPC expansion in a TLR-independent manner [42]. Pathogen-associated molecular patterns or PAMPs released in this model include the TLR agonist lipopolysaccharide and the STING agonist 3′,3′ cyclic-di-GMP (c-di-GMP). This bacterial second messenger is structurally similar to the endogenous cGAS-generated cGAMP [43]. Similar to cGAMP, bacterial c-di-GMP binds to and activates STING. Kobayashi et al. demonstrated that in vivo c-di-GMP treatment promotes multipotent progenitor expansion in a manner dependent on STING, IRF3/7, and IFNAR [44•]. In contrast, long-term HSC levels were diminished upon c-di-GMP treatment, which was dependent on STING and NF-κB signaling but independent of IFNAR. These findings suggest that STING activation elicits distinct responses based on the cellular context. Although this work supports a role for infection-based STING activation in regulating HSPC behavior, a role for endogenous cGAS-STING signaling was unexplored.

In addition to dsDNA, cGAS can be activated by RNA:DNA hybrids [45]. RNA:DNA hybrids along with ssDNA are components of R-loops, which are nucleic acid structural variants that are essential for many cellular processes, such as transcriptional regulation, genomic stability, and immunoglobulin class switching [46]. R-loop imbalance is associated with numerous human diseases, including cancer and immune disorders. Our group recently demonstrated that R-loop imbalance can expand HSPCs [47••]. Using zebrafish, we showed that DEAD-box helicase 41 (DDX41), a factor mutated in inherited adult-onset myelodysplastic syndrome (MDS), suppresses R-loop accumulation. When DDX41 levels fall, R-loop levels rise, and trigger activation of the cGAS-STING pathway, which boosts HSPC production. R-loops induced TBK1 phosphorylation, ISG expression, and NF-κB activation, consistent with the findings of c-di-GMP stimulation. Our findings, showing cGAS-mediated HSPC expansion in DDX41 mutant animals, indicate a link between cGAS activation and clonal hematopoiesis disorders such as MDS.

Although a role for cGAS-STING in the aging HSC compartment has not been investigated, several recent discoveries indicate that it is worth exploring. Aging is associated with low-dose chronic inflammation — inflammaging — that contributes to age-related decline in HSC function and is also linked to aberrant HSPC expansion observed in clonal hematopoiesis [48–54]. It is posited that lifelong accumulation of damaged cellular components that stimulate cGAS and STING such as micronuclei, cytosolic chromatin fragments, damaged nuclear DNA, cytosolic mtDNA, and nuclear envelope disruptions are major contributors of inflammaging [55–58]. Mitochondrial aberrations and mtDNA transfer are linked to HSC dysfunction with age, but the full underlying molecular and cellular mechanism remains unclear [59]. Future exploration into cGAS-STING as mediators of HSPC defects downstream of mitochondrial disruption is warranted.

Cellular senescence is another major cellular hallmark of aging [60]. Recent evidence demonstrated that cGAS is activated by senescence-associated damaged cellular components [33, 61]. Senescence-activated cGAS starts a feed-forward loop that stimulates STING-mediated senescence-associated secretory phenotype (SASP), which promotes paracrine senescence. Loss of cGAS can abrogate senescence in cell culture models [33, 61], and recent in vivo studies indicate that age-associated phenotypes, such as decline in immune cell function and cancer development, also appear to be STING-mediated [62]. As cGAS-STING pathway signal strength elicits different responses, it will be interesting to decipher the different outcomes from acute versus low-dose chronic pathway activation on HSCs and other stem cell populations that decline with age.

New data also link the cGAS-STING pathway in skin stem cells and homeostasis. Endogenous retroviruses (ERV) produced upon microbiota stimulation in the skin and in purified keratinocytes elicit activation of the cGAS-STING pathway and establish the communication between the microbiota and the keratinocytes required for the induction of homeostatic T cell responses to a skin microbe. Mild ERV expression has a positive effect on skin tissue repair and promotes immune fitness. However, hyper-elevated levels of ERVs due to high-fat diet resulted in skin inflammation [63]. Another recent study showed that hair follicle stem cells from patients with hidradenitis suppurativa, a chronic, relapsing, inflammatory skin disease, are disturbed [64]. The patient samples had an expansion of proliferating hair follicle progenitors and decreased numbers of quiescent hair follicle stem cells due to elevated replication stress that triggered excessive STING-mediated type I IFN production. These data are highly reminiscent of the effect of cGAS-STING activation in HSPC, suggesting a potential similarity in the response of somatic stem cells to DNA-sensing pathway induction.

### Mechanism of cGAS Control

cGAS is activated by dsDNA that is present in all cells, so how does it remain inactive? It was long thought that cGAS was mostly localized to the cytoplasm, such that it was only activated upon the entry of foreign DNA into the cell. In recent years that model was challenged when it was discovered that cGAS is largely confined to the nucleus [65, 66]. How does an enzyme that is activated by DNA stay silent in the sea of genomic DNA in the nucleus? Several seminal papers published in 2020 uncovered the answer to this conundrum. Within the genome, cGAS is broadly localized but enriched in heterochromatic, nucleosome-rich regions [67]. It was found that tight binding of cGAS to the histone H2A/H2B dimer in a nucleosome suppresses its activity [66, 68–71]. That said, purified chromatin can stimulate cGAS activity, potentially due to cGAS interaction with dsDNA found in nucleosome-free regions of chromatin [39, 72]. Beyond nucleosome protection of dsDNA, several non-nucleosome chromatin factors regulate cGAS accessibility to nucleosome-poor genomic regions [73]. For example, Barrier-to-autoantigen 1 (BAF), another sequence-independent dsDNA binding protein, displaces cGAS from dsDNA in a competitive fashion [65]. Chromatin architecture is also suggested to modulate cGAS activity as there has been a new link between linker histones and cGAS-driven type I IFN gene expression [74]. The biophysical properties of STING can also control cGAS activation. A recent study showed that STING can condensate into puzzle-like structures in the ER via liquid–liquid phase separation of soluble proteins. These biomolecular condensates limit the immune response to pathogen invasion by sequestering STING and TBK1 [75]. Formation of biomolecular condensates was dependent on cGAMP levels suggesting a level of feedback regulation to prevent pathway overactivation.

Another level of cGAS regulation was identified in murine LT-HSCs that express high levels of cia-cGAS, a circular RNA that interacts with cGAS in the nucleus [76••]. Binding of cia-cGAS to cGAS inhibits its synthase activity and prevents cGAS recognition of self DNA. Similar to c-di-GMP-treated mice, cia-cGAS-deficient animals showed elevated expression of type I IFNs, increased HSPC proliferation, higher numbers of LSK HSPCs, and lower numbers of LT-HSCs. In these mice, HSCs eventually exhaust due to chronic cGAS activation, and subsequent type I IFN overstimulation, thus leading to a bone marrow failure phenotype. All current data support the need for tight regulation of STING-mediated pathways for cellular and tissue homeostasis.

### RNA Sensing-RLR Pathway

RLRs are a family of cytoplasmic receptors that include RIG-I, melanoma differentiation-associated protein 5 (MDA5), and laboratory of genetics and physiology 2 (LGP2) [21, 77]. All RLRs have a helicase domain and carboxy-terminal domain (CTD) while RIG-I and MDA5 also include two caspase activation and recruitment domains (CARDs) that are mainly responsible for signal mediation. RNAs activate RIG-I and MDA5 which then oligomerize through the CARD domains with the mitochondrial antiviral-signaling protein (MAVS) and create a signaling platform leading to activation of kinases like TBK1 and IκB kinase ε (IKKε) (Fig. 1). Ultimately translocation of IRF3, IRF7, and NF-κB to the nucleus lead to the induction of an IFN response and the secretion of proinflammatory cytokines [21].

### RIG-ing Stemness

In recent years more and more evidence suggest that RLRs play critical roles in physiological or pathological processes that are not directly related to immune responses. Recent evidence from our lab suggested a role for RIG-I-like receptors in HSC formation during development [78••]. During development, we found that RIG-I and MDA5 display low-level activity during EHT and activate NF-κB signaling that enhances HSC formation. LGP2 on the other hand restricts NF-κB activation and the function of RIG-I and MDA5, thus acting as a negative regulator of inflammatory signaling during HSC development. A potential source of activation of RIG-I and MDA5 comes from TE transcripts that are expressed during EHT. In adulthood, it was shown previously that RIG-I is differentially expressed between multipotent and myeloid committed progenitors [79]. Moreover, a potential antileukemic effect of RIG-I was shown in acute myeloid leukemia (AML) cells via competitive inhibition of the Src/AKT interaction. Indeed, decreased RIG-I leads to hyperactivity of Src family kinases and subsequent AKT activation [80]. This function of RIG-I is not primed by foreign RNA. We recently illustrated the function of MDA5 in hematopoietic regeneration after chemotherapy [81••]. We found that the transcription of TEs progressively increases in HSCs after chemotherapy. TE transcripts activate MDA5 to induce an inflammatory response that is necessary for HSCs to exit quiescence and proliferate in order to replenish differentiated blood cells that have been eliminated by chemotherapy. Indeed, overexpression of TE copies or knockdown of LINE1 increased or decreased cycling of HSCs in vitro, respectively. TE transcriptional upregulation is also observed in HSCs after irradiation while inflammatory signaling plays instrumental roles in regulating their expression [82•]. Interestingly, TE upregulation has been instrumental in cancer therapy upon treatment with demethylating agents. In these cases, demethylating agents increased transcription of TEs that were sensed by MDA5 thus leading to induction of inflammatory responses and subsequently to cell death [83–85]. Similarly, upregulation of LINE1 upon loss of MPHOSPH8 or MPP8 (M-Phase Phosphoprotein), a member of the HUSH complex, resulted in DNA damage and tumor regression or arrest [86]. The implication from these studies is that triggering RNA-sensing pathways could be instrumental for some cancer immunotherapeutic approaches.

An important role of MDA5 was also identified during the mesenchymal-to-epithelial transition (MET) during induced pluripotent stem cell (iPSC) reprogramming [87]. Loss of ADAR1-mediated RNA editing led to increased activation of MDA5 that disrupted MET. Mechanistically, they showed that ADAR1 editing of transmembrane-encoding dsRNAs is crucial to prevent MDA5 activation and to promote PERK-dependent unfolded protein response that drives MET. RLRs also play a role in mesenchymal stem cell survival [88].

LGP2, the third member of the RLR family whose function in inflammatory pathways remains controversial, has been shown to promote cell survival and fitness of CD8 + T cells after infection — thus, underscoring a possible role under physiological conditions [89]. In contrast, our results showed that LGP2 acts as a negative regulator of HSPC formation during development [90]. Additionally, MAVS, the downstream adaptor of the RLR pathway, is important for mitochondrial homeostasis since it acts as a potential receptor for mitochondria-associated autophagic signaling [91].

### Mechanism of RLR Stimulation Control

The high-affinity ligands for RLR receptors are viral RNAs with specific characteristics that cannot be found in host RNAs, thus providing the first layer of control of RLR and especially RIG-I activation. Additionally, the CARD domains of RIG-I interact with a domain located between the helicase domains keeping RIG-I in a closed conformation, thus preventing activation by ligands [21]. RNA binding also plays a role in controlling RIG-I signal and especially its attenuation. For example, exosome transfer of the non-coding 5′-PPP-containing *RN7SL1* RNA produced in stromal cells triggers RIG-I activation in adjacent breast cancer cells, which induces the production of a cyclic long non-coding RNA that binds to RIG-I and attenuates the signal [92]. Competition of a host-derived, IFN-inducible long noncoding RNA, lnc-Lsm3b with viral RNA for RIG-I binding is another example [93].

Another important regulator of RLR activity is RNA editing by ADAR1. Multiple publications have shown, especially for MDA5, that editing of RNAs by ADAR1 reduces the immunogenicity of these RNAs [27, 29]. Editing of RNA Polymerase II-transcribed *Alu* elements by human ADAR1 blocked translational shutdown by inhibiting hyperactivation of RLRs such as MDA5 or protein kinase R (PKR) in neuronal cells [27]. Additionally, Ahmad et al. showed that the amount of RNA is critical to control pathway activation as increasing the amount of RNA disrupted MDA5 signaling, presumably due to interference with filament formation [94]. RNA modifications also play a role. It was recently shown that m^6^A RNA modification of TEs reduced their half-life, and thus diminished the chances of activating the RNA-sensing pathway [95].

### Other Sensors and Stimuli

As mentioned earlier, besides cGAS and RLRs many other sensors exist. Since current findings support a role for nucleic acid sensors in developmental processes, it is natural to posit that other sensors, possibly activated by diverse stimuli, could have similar roles. Indeed, it was recently shown that loss of H3K9 methylation in G9a-deficient animals results in ERV induction leading to an AIM2-mediated inflammatory response that is devastating for mammary gland development [96]. Since these experiments were performed in the G9a knockout background, it is interesting to speculate that basal expression of ERVs may play a functional role in mammary gland development, but massive TE transcriptional upregulation impairs mammary gland development.

Metabolic regulation of NLRP3 activity and subsequent activation of interleukin-1-beta (IL-1β) was also shown to regulate HSPC formation during development, thus pinpointing an interesting interplay between metabolism and inflammation in the regulation of cell fate decisions [97]. NLRP3 is activated by a variety of stimuli like ATP and uric acid but also mitochondrial DNA, pointing to diverse stimuli that can fine-tune the function of this receptor in physiological processes. It was also recently shown that human NLRP1 is a viral sensor of dsRNA, suggesting that NLRP1 may well sense endogenous dsRNA from TEs or other types of RNAs [98]. Of course, many other sensors could play potential roles in stem cells. For example, TLRs are known to be critical for HSC functions but their potential roles in sensing endogenous ligands remain to be fully explored [99].

## Conclusions

The utility of RLRs and cGAS-STING signaling is best characterized in response to accumulation of danger signals from infectious or endogenous sources. Although RLRs and cGAS-STING are non-essential genes, animals and humans with mutations in RLRs, cGAS-STING, or downstream signaling components are prone to immune dysregulation, such as autoimmunity and infectious susceptibility [100]. Additionally, antiviral immunity plays a central role in stem cell gene engineering [101]. The recent work from our groups and others indicate that these sensing pathways could also titrate stemness properties. An innate quality of all stem cells is their ability to sense and adapt to the changing local and systemic environments while faithfully sustaining themselves and their differentiated progeny. RLRs and cGAS-STING are ancient pathways for evading foreign invaders that then evolved to cope with intrinsic damage. Similarly, we posit that the pathways adapted to modulate stem cell decisions are related to the stimulant, signal strength, and cellular context. For example, it is tantalizing to speculate that the massive nucleosomal shifting associated with cell fate transitions could stimulate low levels of cGAS activation that enforces cell fate states, similar to what was suggested for RNA sensors such as RIG-I and MDA5 [78••]. Additionally, the results discussed in this review hint to the fact that high cellular plasticity triggers nucleic acid sensors. Hence, such mechanisms may be critical during aging or disease. It will be interesting to further investigate which endogenous ligands activate these receptors in stem cells in physiological and stress-induced contexts. TEs are gaining much attention in development, disease, and aging, but what families of TEs and which of their properties are involved still remains heavily understudied. Not to mention that a plethora of other RNAs could still stimulate nucleic acid sensors. Phenomena like hypertranscription and pervasive transcription are prevalent in cell fate changes during development and stress responses, but their importance or purpose during these times still remains to be elucidated [102, 103]. It is possible these events result in nucleic acid sensor activation. Taking into account that more and more stimuli activate DNA/RNA sensors, an emerging idea is that these receptors act as “buffers” of cellular activity during fate transitions, the response of which depends on intrinsic or extrinsic factors. Detailed studies on the direct impact of different stimuli for different sensors are still needed to fully understand this phenomenon. Is it possible that sensors sense everything? Probably not, but there is much more to discover in nucleic acid sensor biology, especially in the realm of stem cell regulation.
